# Association of Dietary Selenium Intake with Type 2 Diabetes in Middle-Aged and Older Adults in China

**DOI:** 10.3390/nu16142367

**Published:** 2024-07-21

**Authors:** Fangyuan Li, Xi Hong, Huijun Wang, Weiyi Li, Lili Chen, Liusen Wang, Boya Zhao, Shaoshunzi Wang, Hongru Jiang, Zhihong Wang

**Affiliations:** 1 Office of National Nutrition Plan, National Institute for Nutrition and Health, Chinese Center for Disease Control and Prevention, 27 Nanwei Road, Beijing 100050, China; lify@ninh.chinacdc.cn (F.L.); hx2015xy@163.com (X.H.); wanghj@ninh.chinacdc.cn (H.W.); liwy@ninh.chinacdc.cn (W.L.); chenll@ninh.chinacdc.cn (L.C.); wangls@ninh.chinacdc.cn (L.W.); zhaoby@ninh.chinacdc.cn (B.Z.); wangssz@ninh.chinacdc.cn (S.W.); 2 Key Laboratory of Public Nutrition and Health, National Health Commission of the People’s Republic of China, Beijing 100050, China

**Keywords:** dietary selenium intake, type 2 diabetes, middle-aged and older adults, China

## Abstract

The relationship between distinct dietary selenium intake and type 2 diabetes (T2D) is still a topic of uncertainty. This study examined the relationship between dietary selenium intake and T2D risk among middle-aged and older Chinese adults. Dietary selenium intake was assessed through three 24 h recalls, using data from the China Health and Nutrition Survey. To investigate the relationship and the potential dose–response pattern between selenium intake and the likelihood of developing T2D, we employed both the restricted cubic spline analysis and the Cox proportional hazards model as our analytical tools. A cohort of 5970 participants aged ≥ 50 years was followed for an average of 5.44 years. The results revealed a V-shaped correlation between selenium intake and T2D risk, with the lowest risk observed at approximately 45 µg/day. Below this level, the risk decreased with an increasing selenium intake, while the risk increased between 45 and 100 µg/day. No significant association was found beyond 100 µg/day. These findings suggest that both low and high selenium consumption may increase T2D risk, highlighting the importance of maintaining a balanced selenium intake for T2D prevention.

## 1. Introduction

Type 2 diabetes (T2D) continues to pose a significant public health challenge, representing a growing threat to global well-being. It is estimated that the number of adult patients with diabetes in developed and developing countries will increase by 20% and 69%, respectively, from 2010 to 2030 [1]. According to the latest International Diabetes Federation (IDF) report, nearly 783 million people will be diagnosed with diabetes worldwide by 2045 [2]. The prevalence of diabetes among Chinese adults witnessed a notable rise, escalating from 10.9% in 2013 to 12.4% in 2018 [3]. With the increasing aging of the population in China, the prevalence of diabetes in the elderly has increased significantly. According to the data of the International Diabetes Alliance in 2019, the number of elderly patients (≥65 years old) with diabetes in China is about 35.5 million, ranking first in the world, accounting for 1/4 of the global elderly patients with diabetes, and showing an upward trend [4]. In addition to genetics, exercise, and environmental risk factors, dietary nutrient intake may also be linked to the development of T2D [5,6].

Selenium, which is prevalent in animal-derived foods, cruciferous vegetables, and cereals, is a crucial trace element necessary for the human body [7]. In human beings, the nutritional functions of selenium are achieved by selenoproteins, which have a wide range of anti-inflammatory and antioxidant effects [8]. As suggested by experimental models, oxidative stress can reduce insulin secretion and contribute to the development of T2D [9,10]. However, the relationship between dietary selenium intake and T2D remains inconclusive [11]. Some studies, including two meta-analyses and a prospective study, have suggested a positive correlation between selenium intake and T2D risk [12,13,14], while findings in another meta-analysis failed to support this association [15]. Additionally, other meta-analysis findings have indicated the potential beneficial effects of selenium on fasting insulin levels and insulin sensitivity [16]. A dose–response analysis further revealed that daily selenium intake exceeding 60 micrograms may be linked to an increased risk of T2D [13].

However, the link between dietary selenium intake and T2D remains complex and inconclusive. Previous research has often overlooked middle-aged and older individuals, who are at increased risk of T2D, and many studies have been cross-sectional, limiting definitive conclusions [17]. Consequently, this study delved into the correlation between dietary selenium intake and diabetes among individuals aged 50 and above.

## 2. Materials and Methods

### 2.1. Study Design and Subjects

This study utilized data from the China Health and Nutrition Survey (CHNS), a long-term longitudinal study that began in 1989 and has completed 11 rounds of follow-up evaluations. Through the implementation of a stratified multistage cluster random sampling technique [18], the survey encompassed 15 provinces (autonomous regions, municipalities), specifically Beijing, Shaanxi, Liaoning, Heilongjiang, Shanghai, Jiangsu, Zhejiang, Shandong, Henan, Hubei, Hunan, Guangxi, Chongqing, Guizhou, and Yunnan. During each iteration of the investigation, a meticulous collection of comprehensive health and nutrition data was undertaken, spanning individual, family, and community levels, ensuring a cohesive and consistent dataset for the same population cohort across all rounds. This included demographics, lifestyle factors (including physical activity, smoking and drinking habits, etc.), dietary habits, economic status, and community conditions, among others. Detailed methodologies for these specific investigations can be found in the relevant literature [19,20,21]. Additionally, the survey protocol was augmented in 2009, 2015, and 2018 to include the collection and testing of blood samples.

**Figure 1:**
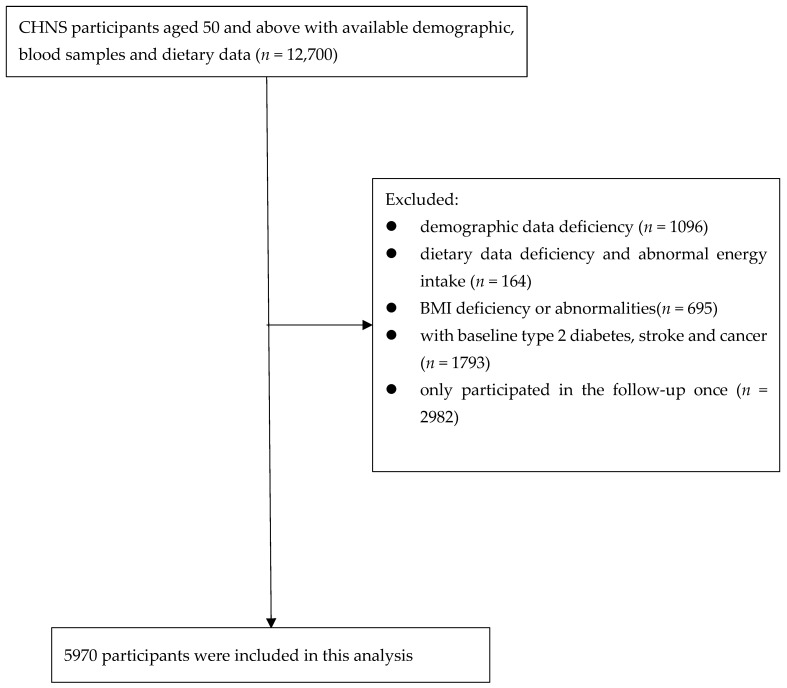
Flowchart of subjects.

Adults aged 50 and older who participated in the 2009, 2015, and 2018 surveys were specifically selected. We set the subject exclusion criteria as follows: demographic information deficiency (*n* = 1096), dietary data deficiency and abnormal energy intake (>6000 kcal/day or <800 kcal/day for men; >4000 kcal/day or <600 kcal/day for women) (*n* = 164), BMI deficiency or abnormalities (<14 kg/m^2^ or >45 kg/m^2^) (*n* = 695), baseline T2D, stroke, or cancer (*n* = 1793), participated in the follow-up only once (*n* = 6113). Eventually, 5970 subjects were included in this research (Figure 1). This project adhered to rigorous ethical standards, having secured approval from the Ethics Review Committee of the Institute of Nutrition and Health at the Chinese Center for Disease Control and Prevention (Review number: 2015-017). Prior to the survey, all respondents provided informed consent.

### 2.2. Evaluation Indicators

#### 2.2.1. Dietary Nutrients

To ensure comprehensive and robust data collection for this study, the information was gathered through rigorous face-to-face surveys. The collection of personal food consumption data relied on an exhaustive 3-day, 24 h dietary review methodology, encompassing two weekdays and a weekend day, providing a thorough evaluation of individual dietary patterns and habits. This approach aimed to capture the usual dietary intake of individuals accurately, considering day-to-day variations in eating patterns. Additionally, information regarding the usage of condiments and edible oils was meticulously gathered through the implementation of household weighing method. These data were then meticulously allocated to each individual participant, proportional to their individual energy consumption within the household, providing a precise estimation of their dietary selenium intake from these sources. Using the “Chinese Food Ingredient Table” as a reference, the collected consumption data related to various foods, seasonings, and edible oils were converted into nutrient intake estimates [22]. The computation of average daily nutrient intake was founded upon the total person-days recorded throughout the entire survey period.

To mitigate the potential confounding influence that dietary alterations subsequent to disease onset might exert on the relationship between dietary selenium intake and diabetes, a tailored strategy was devised and implemented. The dietary selenium intake value observed at the end of the disease period was excluded from the analysis. This was carried out to prevent any bias that might arise from alterations in dietary habits following the diagnosis or development of the disease. In addition, energy-standardized dietary selenium intake was used to control the impact of total energy intake [23].

#### 2.2.2. Diagnostic Criteria for Diabetes

Blood samples were collected via venipuncture after 8–12 h of fasting. Both fasting plasma glucose (FPG) and hemoglobin A1c (HbA1c) levels were promptly measured following sample collection. FPG was determined using the glucose oxidase–phenol and aminobenzene method, while HbA1c was determined by high-performance liquid chromatography. Type 2 diabetes is defined as having a FPG concentration ≥ 7.0 mmol/L and/or HbA1c mass fraction ≥ 6.5%. Furthermore, those diagnosed with diabetes and currently undergoing treatment are also considered as patients with type 2 diabetes.

#### 2.2.3. Evaluation of Confounding Factors

This study gathered an array of pertinent data through face-to-face interviews conducted by highly trained and qualified investigators. Utilizing a standardized questionnaire, the research team meticulously documented demographic information, lifestyle factors (encompassing smoking status, alcohol consumption habits, physical activity levels, among others), dietary habits, and other crucial variables. Educational level was classified into three groups: primary school or below, middle school, and college or above. Residence was categorized as urban or rural. Household per capita annual income was categorized into three groups: low (<6128.13 yuan), medium (6128.13–17,611.37 yuan), and high (>17,611.37 yuan). The participants were divided into three age groups: 50–64 years, 65–79 years, and 80 years and above. Smoking status and alcohol consumption within the past year were categorized into binary variables: ‘yes’ or ‘no’. Meanwhile, physical activity levels were comprehensively assessed, encompassing leisure-time physical activity, transportation-related physical activity, occupational physical activity, and household-related physical activity. The physical activity volume (MET h/week) was assessed by multiplying the metabolic equivalent (MET) of each activity by the weekly duration of participation in various physical activities (h/week) and was further categorized into three groups based on tertiles: low (<96.24 MET h/week), medium (96.24–<228.96 MET h/week), and high (≥228.96 MET h/week). BMI was calculated as weight (kg) divided by height (m) squared and classified into three groups: <18.5 kg/m^2^, 18.5–<24.0 kg/m^2^, and ≥24.0 kg/m^2^.

### 2.3. Statistical Analysis

Data collation and analysis were conducted using R software (version 4.0.0) and SAS software (version 9.4). Descriptive statistics were strategically used to provide a comprehensive overview of research variables. Quantitative variables are concisely represented as mean values with standard deviations. Qualitative variables are presented in percentage form. To examine the demographic profile of participants across different dietary selenium intake levels, chi-square tests and analysis of variance were employed. Additionally, selenium intake was grouped into quintiles, and diabetes outcomes were tracked from cohort entry to disease onset. To delve into the intricate relationship between dietary selenium intake and diabetes risk, this study leveraged the power of multivariate Cox proportional hazard regression modeling. Furthermore, the restricted cubic spline (RCS) model was employed to trace the changing trend in diabetes risk as dietary selenium intake increased. Statistical significance was determined based on a *p*-value threshold of less than 0.05.

## 3. Results

### 3.1. Baseline Characteristics

A total of 5970 subjects were enrolled in this study. The median dietary selenium intake was 41.04 µg/day. The baseline demographics and characteristics of the study subjects were categorized into five distinct quintiles according to their dietary selenium intake, as detailed in Table 1. Compared with the subjects in Q5, individuals with lower dietary selenium intake tended to be female, elderly, low-income, low-educated, living in rural areas, non-smoking, non-drinking, not overweight or obese, and had a lower energy intake. Additionally, no significant difference in the level of physical activity was observed among different dietary selenium intake groups.

### 3.2. Effect of Dietary Selenium Intake on the Risk of T2D

The interaction between gender and dietary selenium intake and diabetes risk was analyzed, and the results demonstrated no interaction (*p* = 0.94). Subsequently, gender was incorporated into the model as an essential confounding variable to refine the analysis. The total follow-up duration was 32,479.06 person-years, averaging 5.44 years per individual. During this period, 718 cases of diabetes were identified, resulting in an incidence rate of 12.03%. The Cox proportional risk regression model analysis results showed (Table 2) that when taking the Q1 group as a reference, the risk of diabetes of the subjects in Group Q3 decreased by 32% (HR = 0.68, 95% CI: 0.53~0.87) in Model 1 and Model 2, while in Model 3, the risk of diabetes in Group Q3 was reduced by 26% (HR = 0.74, 95% CI: 0.57~0.95). After further adjustment for all confounding factors, participants in Group Q3 experienced a 27% reduction in the risk of developing diabetes compared to those in Group Q1 (HR = 0.73, 95% CI: 0.56~0.94). In all models, the difference in selenium intake between Q2, Q4, and Q5 groups and the risk of diabetes was not statistically significant. In addition, no significant difference in the linear trend test was detected in all models (*p* > 0.05).

### 3.3. Dose–Response Relationship between Dietary Selenium Intake and T2D

**Figure 2:**
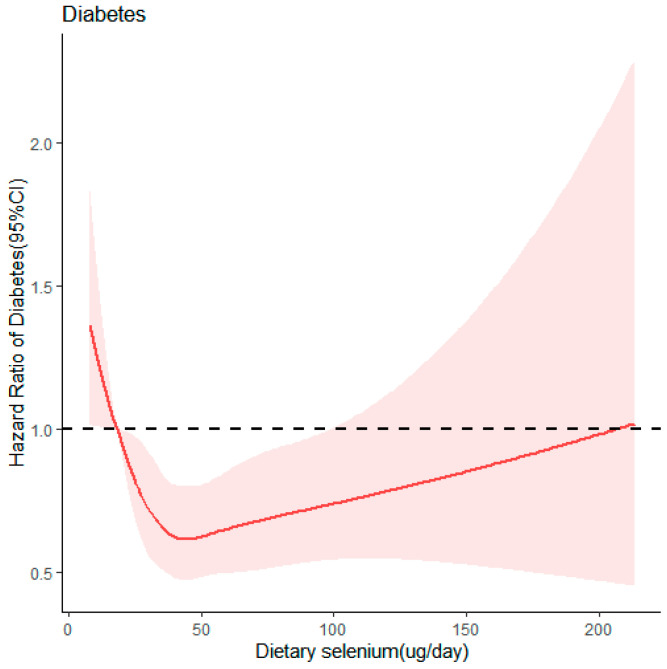
Dose–response relationship between dietary selenium intake and risk of T2D. Restricted cubic spline of association between dietary selenium intake and Type 2 diabetes. The red-shaded area represents the 95% confidence interval (CI).

The outcomes of the restricted cubic spline model analysis, as depicted in Figure 2, revealed an overall statistically significant (*p* < 0.05) and nonlinear correlation between dietary selenium intake and T2D risk. The analysis revealed a V-shaped correlation when using the 5th percentile of dietary selenium intake (18.05 µg/day) as the reference value. Below 45 µg/day of dietary selenium intake, the risk of diabetes decreased as selenium intake increased, reaching its lowest point at 45 µg/day. However, between 45 and 100 µg/day, the risk gradually increased. When dietary selenium intake surpassed 100 µg/day, the correlation between selenium consumption and diabetes risk was not statistically significant.

## 4. Discussion

This research examined the relationship between dietary selenium intake and T2D among middle-aged and older adults, drawing upon longitudinal data sourced from the CHNS in China. This forward-looking cohort study encompassed 5970 participants, with each individual being followed up on for an average duration of 5.44 years. The findings indicated that participants with dietary selenium levels ranging between 38.66 µg/day and 43.40 µg/day exhibited the lowest risk of T2D compared to those in the lowest quintile of dietary selenium levels. The current analysis unveiled a nonlinear dose–response relationship between dietary selenium intake and T2D, characterized by a V-shaped correlation. Both insufficient and excessive levels of selenium were associated with an increased risk of T2D. Dose–response analysis indicated that participants with dietary selenium levels of around 45 µg/day had the lowest risk of T2D. Notably, the prevalence rate of T2D in this study population was 12.03%, slightly higher than the estimated level reported by the CHNS survey in 2009 (7.5%) [24].

Some studies have proposed a positive correlation between selenium intake and diabetes [25], suggesting that excess selenium may pose a risk. In contrast to NHANES, where the median lowest quartile selenium intake was reported as 70.6 µg/day (similar to the upper quartiles in our study), our population had a generally lower dietary selenium intake. A cohort study from Brazil presented no significant correlation between dietary selenium consumption and the incidence of T2D. It is noteworthy that the median energy-adjusted selenium intake in this study was 143.5 µg/day [26]. Similarly to this study, our study shows that when selenium intake exceeded 100 µg/day, the association between dietary selenium intake and diabetes risk ceased to be statistically significant. A meta-analysis revealed that both relatively low serum selenium concentrations (below 97.5 µg/L) and excessively high levels (above 132.5 µg/L) were associated with an elevated prevalence of T2D. The observed trend of a more pronounced increase in T2D incidence among individuals with high selenium levels, coupled with findings from other studies demonstrating significantly lower plasma selenium concentrations in diabetic patients, underscores the complex and nonlinear nature of the relationship between T2D and selenium levels [27].

According to OgawaWong et al. [26], the association between selenium and T2D follows a U-shaped curve, indicating T2D occurrence with either insufficient or excessively high Se levels. Similarly, in this study, it was observed that dietary selenium levels were associated with T2D risk in a V-shaped manner, where both insufficient and excessive levels were linked to increased risk. Dose–response analysis revealed that participants with dietary selenium levels of around 45 µg/day had the lowest T2D risk, which closely aligns with the recommended dietary selenium intake of 50 µg/day in China [28]. However, the recommended daily intake of selenium varies by geographical region and by different authoritative bodies. For instance, the US Department of Agriculture suggests a recommended daily allowance (RDA) of 55 µg, whereas the European Food Safety Authority (EFSA) recommends an RDA of 60 µg for women and 70 µg for men. Additionally, the International Food and Nutrition Board suggests an average daily intake of 40–70 µg/day for men and 45–55 µg/day for women [29]. A meta-analysis also suggests that a daily intake of selenium above 60 µg may increase the risk of T2D [13]. These differences may be attributed to a multitude of factors, including gender, age, pregnancy, breastfeeding, and geographical location. The median selenium intake in our study population was lower than that reported in some Western populations, possibly reflecting differences in dietary habits and soil selenium content. The lack of a significant association between selenium intake and T2D beyond 100 µg/day in our study contrasts with some previous reports, highlighting the need for region-specific recommendations. The variability in the recommended daily selenium intake across different authorities underscores the complexity of setting universal guidelines.

The complex interplay between selenium and T2D mellitus is multifaceted and involves both protective and potentially detrimental effects. Selenium, with its antioxidant properties, can enhance GPX1 expression and activity, thereby mitigating oxidative stress and inflammation in islet β-cells, potentially offering protection against T2D [30,31,32]. However, some studies have revealed that excessive selenium exposure, particularly through supplementation, may elevate the risk of T2D by augmenting hepatic Sepp1 production, a known inducer of insulin resistance [12,33,34,35]. Given selenium’s essential role in selenoprotein function and insulin signaling pathways, its management in diabetes is crucial [36,37,38]. Yet, the absorption of selenium is influenced by numerous factors such as age, physiological state, selenium form and quantity, nutritional status, medical conditions, and genetic predispositions [39,40]. Notably, different selenium compounds exhibit varying absorption efficiencies, with selenomethionine and selenate demonstrating superior gut absorption compared to others [41]. This disparity between intake and absorption, particularly in the elderly, underscores the need for cautious monitoring. Furthermore, studies have shown that selenium supplementation leads to an increase in both absorbed selenium and whole blood selenium concentrations, which persist for several months post-cessation [42]. Indeed, dietary selenium intake can serve as valuable, non-invasive biomarkers for assessing selenium status and exploring its impact on health outcomes, including T2D. Ultimately, further research is warranted to elucidate the precise molecular mechanisms underlying selenium’s dual role in T2D and to develop targeted strategies for optimal selenium management in diabetes prevention and treatment.

While our study has several strengths, including a large sample size and prospective design, there are also limitations. First, subjects entered the cohort at different times (in 2009 or 2015), serving as relative baselines. Moreover, the dietary surveys conducted in different years might not be entirely consistent due to improvements in living standards or survey methods. Grouping the subjects together might lead to bias, but dietary factors such as income and energy in the model were hereby adjusted to explain some of the bias. Second, dietary data collected through a continuous “3-day, 24-h” dietary review method were adopted to calculate dietary selenium intake, which tended to fail to assess daily fluctuations in dietary intake and investigate long-term dietary patterns and behaviors. Additionally, we did not specifically evaluate malabsorption problems, such as small intestinal bacterial overgrowth, lactose intolerance, delayed orocecal transit time, or modified nutrient absorption like D-xylose, which have been shown to correlate with diabetes, particularly type 2 diabetes mellitus. The potential impact of these malabsorption issues on selenium absorption and diabetes risk warrants further investigation. Non-invasive techniques for assessing malabsorption could be considered in future studies to better understand these relationships.

## 5. Conclusions

In conclusion, this study provides novel insights into the intricate relationship between dietary selenium intake and the risk of T2D among middle-aged and older adults in China. The notable V-shaped association observed between selenium intake and the risk of T2D underscores the critical importance of maintaining an optimal selenium intake range to minimize the risk of diabetes. Our findings highlight the need for tailored interventions aimed at optimizing selenium intake in populations at risk, as both insufficient and excessive selenium intake may increase T2D risk.

## Figures and Tables

**Table 1:** Baseline characteristics according to the quintile of dietary selenium intake.

Baseline Characteristics	Quintile of Dietary Selenium Intake (µg/day)					
	Q1 22.05 (18.05~25.14)	Q2 32.27 (30.03~34.53)	Q3 41.04 (38.66~43.40)	Q4 51.57 (48.55~55.39)	Q5 74.38 (65.96~89.58)	*p*-Value
Age, %						<0.0001
50~64	64.49	73.2	77.81	77.22	80.82	
65~79	31.74	24.12	21.02	21.27	18.26	
80~	3.77	2.68	1.17	1.51	0.92	
Male, %	32.66	42.21	45.98	51.93	60.64	<0.0001
Household income per capital, %						<0.0001
Low (<6128.13 yuan)	42.46	37.94	31.66	30.57	24.04	
Median (6128.13–17,611.37 yuan)	31.16	32.24	34.76	35.51	33.00	
High (>17,611.37 yuan)	26.38	29.82	33.58	33.92	42.96	
Education, %						<0.0001
Primary and below	63.4	53.85	44.56	42.63	35.85	
Middle and high	31.07	35.26	44.3	46.06	48.66	
College and above	5.53	10.89	11.14	11.31	15.49	
Urban, %	28.73	35.68	37.86	40.54	44.89	<0.0001
Never smoked, %	77.72	70.77	71.02	67.84	62.56	<0.0001
Non-drinker in the past year, %	81.07	72.11	69.68	66.5	61.81	<0.0001
Physical activity, %						0.60
Low	33.84	31.24	33.84	34.25	33.42	
Median	31.83	33.75	32.91	33.42	34.84	
High	34.34	35.01	33.25	32.33	31.74	
BMI (kg/m^2^), %						<0.0001
<18.5	6.45	5.36	4.1	3.18	2.26	
18.5~23.9	51.51	52.76	49.25	47.57	45.23	
≥24.0	42.04	41.88	46.65	49.25	52.51	
Energy (kcal/day)	1564.65 ± 492.48	1931.00 ± 589.56	2134.19 ± 602.86	2341.46 ± 654.01	2624.06 ± 764.53	<0.0001

Values are mean ± SD for continuous variables and percentage for categorical variables.

**Table 2:** Multivariate Cox proportional risk regression analysis of dietary selenium intake to type 2 diabetes [HR (95% CI)].

Model	Quintile of Dietary Selenium Intake (µg/day)					*p* Trend
	Q1	Q2	Q3	Q4	Q5	
Model 1	1.00	0.87 (0.69, 1.10)	0.68 (0.53, 0.87) *	0.79 (0.63, 1.01)	0.84 (0.66, 1.08)	0.39
Model 2	1.00	0.87 (0.69, 1.10)	0.68 (0.53, 0.87) *	0.80 (0.63, 1.01)	0.86 (0.67, 1.09)	0.20
Model 3	1.00	0.91 (0.71, 1.16)	0.74 (0.57, 0.95) *	0.84 (0.65, 1.08)	0.90 (0.69, 1.17)	0.43
Model 4	1.00	0.91 (0.72, 1.16)	0.73 (0.56, 0.94) *	0.79 (0.61, 1.03)	0.85 (0.65, 1.11)	0.16

* Comparisons with the lowest quintile (Q1) revealed statistical significance (*p* < 0.05). Model 1 served as the foundation, adjusting for age, education, sex, urban–rural residence, and per capita household income. Model 2 built upon Model 1 by incorporating additional adjustments for alcohol consumption, smoking status, and physical activity levels. Model 3 further refined Model 2 by adjusting for total energy intake. Lastly, Model 4 built upon Model 3 with an additional adjustment for Body Mass Index (BMI).

## Data Availability

The original contributions presented in the study are included in the article, further inquiries can be directed to the corresponding authors.
